# The application of artificial intelligence in health financing: a scoping review

**DOI:** 10.1186/s12962-023-00492-2

**Published:** 2023-11-06

**Authors:** Maryam Ramezani, Amirhossein Takian, Ahad Bakhtiari, Hamid R. Rabiee, Ali Akbar Fazaeli, Saharnaz Sazgarnejad

**Affiliations:** 1 Department of Health Management, Policy and Economics, School of Public Health, Tehran University of Medical Sciences, Tehran, Iran; 2 Health Equity Research Centre (HERC), Tehran University of Medical Sciences, Tehran, Iran; 3 Department of Global Health and Public Policy, School of Public Health, Tehran University of Medical Sciences, Tehran, Iran; 4 Department of Computer Engineering, Sharif University of Technology, Tehran, Iran; 5 School of Medicine, Tehran University of Medical Sciences, Tehran, Iran; 6 School of Public Health, Tehran University of Medical Sciences, Tehran, Iran

**Keywords:** Artificial intelligence, Health financing, Applications

## Abstract

**Introduction:**

Artificial Intelligence (AI) represents a significant advancement in technology, and it is crucial for policymakers to incorporate AI thinking into policies and to fully explore, analyze and utilize massive data and conduct AI-related policies. AI has the potential to optimize healthcare financing systems. This study provides an overview of the AI application domains in healthcare financing.

**Method:**

We conducted a scoping review in six steps: formulating research questions, identifying relevant studies by conducting a comprehensive literature search using appropriate keywords, screening titles and abstracts for relevance, reviewing full texts of relevant articles, charting extracted data, and compiling and summarizing findings. Specifically, the research question sought to identify the applications of artificial intelligence in health financing supported by the published literature and explore potential future applications. PubMed, Scopus, and Web of Science databases were searched between 2000 and 2023.

**Results:**

We discovered that AI has a significant impact on various aspects of health financing, such as governance, revenue raising, pooling, and strategic purchasing. We provide evidence-based recommendations for establishing and improving the health financing system based on AI.

**Conclusions:**

To ensure that vulnerable groups face minimum challenges and benefit from improved health financing, we urge national and international institutions worldwide to use and adopt AI tools and applications.

## Introduction

In the era of AI, with the rapid development of information technology (IT) and various data types, areas such as machine learning (ML) and big data [1] have become essential components of decision-making. An important economic and public health concern worldwide is the financial burden of health care and diseases [2]. AI is a broad term related to advancements that make machines “intelligent” and aims to develop an intelligent and autonomous system. ML enables computers to automatically learn and improve their understanding as a subset of AI without explicit programming. The origin of AI can be traced over fifty years back, its rapidly increasing possibilities in various fields have significantly risen during the past few years [1].

As countries are challenged with rising healthcare costs, they seek to protect their citizens and vulnerable groups from unaffordable high healthcare expenditures and to ensure access to comprehensive, non-discriminatory healthcare services [3]. In recent years, policymakers, national public health managers, and academics have become increasingly concerned with financing public health. Additionally, the high data dimensionality caused by the abundance of risk factors for patient expenditure, such as demographics, diagnoses, comorbidities, etc. makes complex and unpredictable systems [4]. To handle the massive health data, new approaches apply numerous machine learning algorithms. AI applications in healthcare management can help to close the gap between available resources and public healthcare demand. As AI develops, more and more healthcare management applications are being used [5].

Another issue that requires systemic thinking is the stability of insurance funds to control out-of-pocket payments; AI can assist in resolving this issue.

 [6] [7]. The majority of real-world financial systems exhibit nonlinear and uncertain behaviors that may change over time. As a result, there is a greater need to solve highly nonlinear, time-variant challenges, for which AI is gaining popularity [8]. In other words, these new applications are replacing older ones that could only estimate short-term flow by calculating all relevant variables and establishing current and potential future relationships [9].

Hence, the need to gain a detailed overview of new technologies used for health financing is clear, which is essential to support the pathways toward universal health coverage [10]. This article aims to highlight recent applications of the implementation of AI in health financing in light of recent AI advancements and identify potential future research areas.

## Methods

In line with its objectives, we adopted a scoping review framework guided by Arksey and O’Malley’s methodology, including six steps: 1- identifying the research question; 2- identifying relevant studies; 3-selecting included studies; 4-data charting; 5-summarizing and reporting the results; and 6- consultation with experts [11].

Following the first step, we formulated research questions as follows: (1) Which applications of AI in health financing are reported in the literature? (2) What future health financing applications and capabilities can be employed? We then identified relevant studies according to appropriate keywords, and two independent researchers, MR and AB, conducted a comprehensive literature search through three databases: PubMed, Web of Sciences, and Scopus (Table 1). In the third step, two independent authors MR and AB, screened the relevance of titles and abstracts of articles and reviewed the full texts of the relevant articles. We charted the extracted data in the fourth step and collated, summarized, and reported in the fifth step. Finally, we reported the synthesized results to the research team and one external advisor for approval.

### Inclusion criteria for study selection

Inclusion criteria were studies published in English on financing health systems (inclusively or exclusively) from 2000 to 2023. Articles without full-text were excluded.

**Table 1:** Search queries used for target databases

Databases	Query	Initial results
**Scopus**	( ( TITLE-ABS-KEY ( “big data” ) OR TITLE-ABS-KEY ( “data mining” ) OR TITLE-ABS-KEY ( “deep learning” ) OR TITLE-ABS-KEY ( “artificial intelligence” ) OR TITLE-ABS-KEY ( “Expert system” ) OR TITLE-ABS-KEY ( “Intelligent system” ) OR TITLE-ABS-KEY ( “Knowledge system” ) OR TITLE-ABS-KEY ( “Decision Support System” ) OR TITLE-ABS-KEY ( “machine learning” ) ) ) AND ( TITLE-ABS-KEY ( health ) ) AND ( ( TITLE ( financing ) OR TITLE ( financial ) OR TITLE ( “social insurance” ) OR TITLE ( “government budget” ) OR TITLE ( finance ) OR TITLE ( GDP ) OR TITLE ( expenditure ) OR TITLE ( grant ) OR TITLE ( investment ) OR TITLE ( invest ) OR TITLE ( fund ) OR TITLE ( loan ) OR TITLE ( loans ) OR TITLE ( funds ) OR TITLE ( “health insurance” ) OR TITLE ( “strategic purchasing” ) OR TITLE ( “pooling of funds” ) OR TITLE ( “risk pooling” ) OR TITLE ( revenue ) OR TITLE ( tax ) OR TITLE ( “resource allocation” ) OR TITLE ( “purchasing of services” ) OR TITLE ( “revenue collection” ) ) )	599
**Web of Sciences**	(((((((((((((((((((((((TI=(financing)) OR TI=(financial)) OR TI=(“social insurance”)) OR TI=(“government budget”)) OR TI=(finance)) OR TI=(GDP)) OR TI=(Expenditure)) OR TI=(grant)) OR TI=(investment)) OR TI=(invest)) OR TI=(fund)) OR TI=(loan)) OR TI=(loans)) OR TI=(funds)) OR TI=(“health insurance”)) OR TI=(“strategic purchasing”)) OR TI=(“pooling of funds”)) OR TI=(“risk pooling”)) OR TI=(revenue)) OR TI=(tax)) OR TI=(“resource allocation”)) OR TI=(“purchasing of services”)) OR TI=(“revenue collection”)) (((((((((AB=(“Artificial intelligence”)) OR AB=(“machine learning”)) OR AB=(“data mining”)) OR AB=(“deep learning”)) OR AB=(“Expert system”)) OR AB=(“Intelligent system”)) OR AB=(“Knowledge system”)) OR AB=(“Decision Support System”)) OR AB=(“big data”)) AB=(health)	202
**PubMed**	((((((((“big data“[Title/Abstract]) OR (“data mining“[Title/Abstract])) OR (“deep learning“[Title/Abstract])) OR (“artificial intelligence“[Title/Abstract])) OR (“Expert system“[Title/Abstract])) OR (“Intelligent system“[Title/Abstract])) OR (“Knowledge system“[Title/Abstract])) OR (“Decision Support System“[Title/Abstract])) OR (“machine learning“[Title/Abstract]))))) AND ((((((((((((((((((((((((((((financing[Title]) OR (financial[Title])) OR (“social insurance“[Title])) OR (government budget[Title])) OR (finance[Title])) OR (GDP[Title])) OR (Expenditure[Title])) OR (grant[Title])) OR (investment[Title])) OR (invest[Title])) OR (fund[Title])) OR (loan[Title])) OR (loans[Title])) OR (funds[Title])) OR (“health insurance“[Title])) OR (“strategic purchasing“[Title])) OR (“pooling of funds“[Title])) OR (“risk pooling“[Title])) OR (revenue[Title])) OR (tax[Title])) OR (“resource allocation“[Title])) OR (“purchasing of services“[Title])) OR (“revenue collection“[Title])))))))))])	378

### Flow chart of the search strategy

**Fig. 1:**
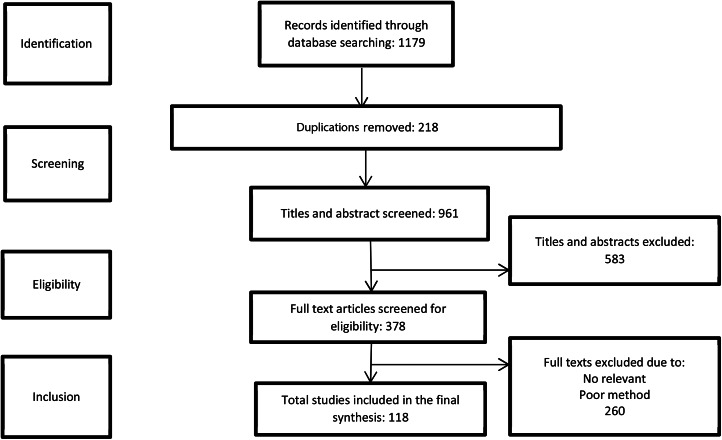
Flow chart of the search strategy

1179 references were identified during the initial search, 218 of which were duplicates, providing 961 articles from the first screen title review. We then excluded 583 articles that did not meet the inclusion criteria, 378 studies met the inclusion criteria for full-text evaluation, and 260 were excluded due to the lack of relevancy or poor methodological quality (Fig. 1). The results of the study are presented in two parts. Initially, we summarize the extracted information, propose a framework, and explain its dimensions.

### Charting data

Two authors, MR and AB, synthesized the data by placing similar codes into categories of AI applications in health financing to develop a conceptual framework and began to create descriptions by synthesizing the extracted information. All authors reviewed and discussed the framework until we reached a final consensus.

## Results

### A Framework for future studies: existing knowledge and future directions

**Fig. 2:**
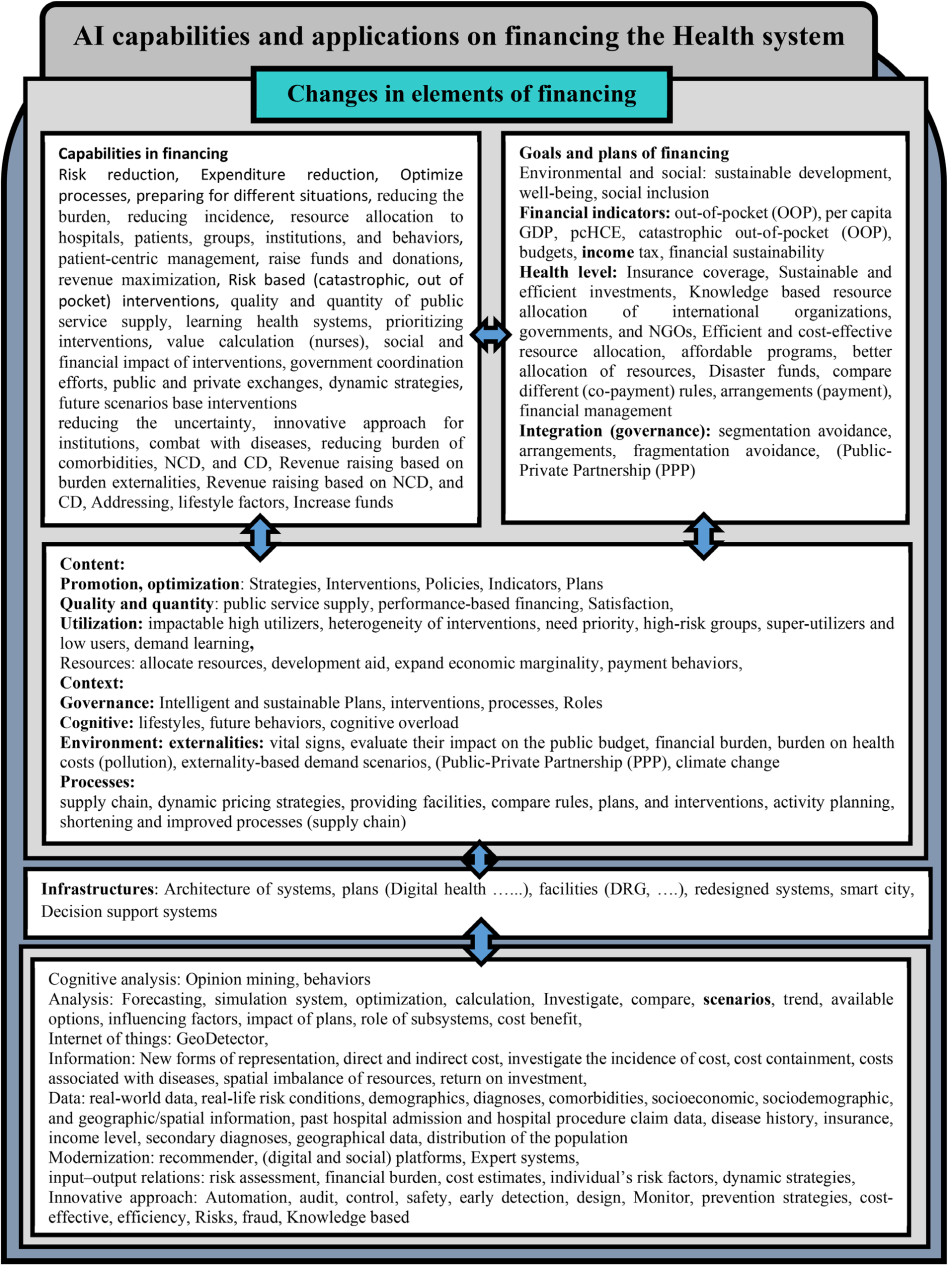
AI applications and capabilities in health financing. (Contemporary studies and future agenda)

As shown in Fig. 2, the proposed framework has five components. The conceptual framework shows that AI can affect health financing with different applications and capabilities.

### AI applications and capabilities

Applications of AI have been used or have a high potential to improve performance in a variety of areas of health financing, including governance, revenue raising, pooling, and strategic purchasing.

### 1-Governance

#### 1-1 process

Studies have discussed various assorted implications and characteristics for applying AI and advanced data management tools [12] in the governance of health financing. At the macro level, AI can help investigate which social determinants of health (SDH) may cause more cost within a specific patient population [13]. One study introduced the development of an ML framework to automate classifying health projects into different global common goods for health (CGH) categories. The study illustrates the feasibility and efficiency of tracking financing for global CGH [14]. Another study used various datasets to investigate the extremes of micro and macro variables and the country flow of funds to develop a general equilibrium model encompassing regional variation in the type of financial friction and calibrating it to measure variation in regional aggregates [15].

One study provides a simplified simulation system framework for emergency material preparation and dispatch with high operating efficiency [16]. Researchers in another study developed a detailed decision support system called “Cost Calculation Tool” for the leading Austrian social occupational insurance institution (AUVA) that investigates total direct and primary indirect costs of individual injury claims from the time of the incident for all injured individuals up to their death [17]. Furthermore, analyzing the healthcare costs data subset will make cost audit and artificial sampling audit possible [18].

Public option mining is another application of AI in different areas that can potentially improve health financing [19]. Representation of financial information, for instance, transforms concurrent financial time series into images [20], forecasting the incidence of medical cases, or predicting the degree of spread of the disease, are among the initial steps in institutional planning that help plan health control strategies and develop intervention programs based on required medical resources and effective resource allocation strategies [21–23].

#### 1–2 Content

In recent years, numerous scientific research studies have focused on sustainable development. The results of AI analysis in one study showed the impact of air pollution change on the acceleration of economic growth in India [24]. AI can also facilitate the efficient monitoring of resource allocation of governments, donors, and development agencies involved in achieving Sustainable Development Goals (SDGs). Future studies can explore the simultaneous social and financial impact of different interventions to improve the efficient use of resources for development and create insights about additional opportunities to substantially impact the well-being of people worldwide [25].

The modernization of the digital economy and financial and economic mechanisms is being facilitated through end-to-end technologies, which promote innovation and enhance the quality of life for the population. AI has the potential to address many of the current challenges faced by nations, such as reducing unemployment, improving public sector efficiency, enhancing environmental conditions, fostering innovation, and increasing national economic competitiveness [26]. These changes will be related to reducing the burden of comorbidities, addressing lifestyle factors such as smoking, and screening for cancers and cardiovascular diseases, that need to be targeted to lower the mortality and cost associated with diseases [27]. One of these innovative approaches adopted by the Global Fund to Fight AIDS, TB, and Malaria (GFATM) to health financing has had significant flow-on effects on the knowledge system that supports efforts to combat the three subject diseases in target countries. By considering itself as part of a more extensive knowledge system, the GFATM can use this influence to build the capacity across that system to learn from implementation and refine its processes accordingly [28].

Clinical and socioeconomic perspectives are suggested to avoid determinants of medical cost and improve healthcare resource utilization [29]. One study investigated the incidence of hemorrhoids and associated risk factors like lifestyle behavioral variables, including body mass index (BMI), alcohol intake, smoking, and exercise [30]. These revolutions help promote healthy lifestyles and disease prevention strategies to strengthen universal health care [31]. Moreover, AI can determine the optimum allocation of resources to the areas needing prioritization for attaining social inclusion goals [31]. Decision-makers can select from several rational resource allocation models depending on the data availability and complexity level. For example, S4HARA (System for HIV/AIDS resource allocation) is a four-step spreadsheet-based model for a rational resource allocation approach. Recommendations of the model are grounded in the cultural, social, and political context [32]. Furthermore, determining the overall performances of healthcare structures based on input-output relations plays a vital role in optimizing resource allocation and investment planning, as it contributes to reducing the uncertainty of future performance [33].

#### 1–3 Context

AI can apply an event-detection approach to extract information about real-world events’ consequences [34, 35]. For instance, the influencing factors of air pollution on government health expenditure and its spatial governance can be explored using a Geographical Detector, (GeoDetector) [36].

Using big data analysis, disabilities can be detected earlier than clinical diagnoses, allowing policymakers to act appropriately to prevent disabilities [37]. Population risk assessment can estimate Socioeconomic costs and indicators [38, 39]. For instance, one study estimated socio-economic burden and co-morbidities by including expenses for outpatient care or hospitalization, medicines, prescriptions, traffic expenses, and lost income paid by the insurer and patients [40]. To forecast macroeconomic variables such as Gross domestic product (GDP) growth, AI has the potential to establish connections between individual economic activities and macro-socioeconomic indicators on various spatial scales, to help understand the current and future state of the economy [41].

### 2- Revenue raising

#### 2 − 1 Content

One study used GDP and population data as independent variables to predict life expectancy [42]. To address the challenge of increasing demand with limited resources, decision-makers need to explore innovative approaches that could help sustain the quality of services provided to the citizens. One study focused on obtaining additional resources for the public health budget directly from citizens (38). With this background, researchers developed an agent-based simulation model as a decision support tool to compare different co-payment rules and evaluate their impact on the public budget and the health expenses of different groups of citizens [43].

Another study analyzed whether the deductible impacts co-pay distribution among different patient groups. The study recommends that patients with chronic and non-chronic conditions of similar age and income should pay the same amount for fairness [43]. Another study showed that in a publicly funded system of care, a community-based program could be utilized from tools such as the Child and Adolescent Needs and Strengths (CANS) to identify clients likely to benefit from established mental health services. The study proposed using ML methods to learn from data collected through the application of Transformational Collaborative Outcomes Management (TCOM) within a human services system, which can be replicated in various contexts [44].

Big data technology can bring new foundations and challenges to tax management, but the premise is that big data thinking needs to be formed [9]. To gain the optimal tax, collecting precise data, or at least capturing the appropriate magnitude of the total value of the sum of negative externalities, is crucial. Thus, governments (national, regional, local, and/or any other political initiatives) need to use big data to crack the code of what goes wrong and identify options for moving forward. [45]. Another study illustrated that determining the ideal meat tax size requires a comprehensive analysis of foresight and big data. This approach can lead to a double benefit: reducing meat production to an optimal level while generating tax revenue. Generated tax revenue should be invested in a political decision that calls for international negotiations. There are various possibilities, such as development aid, disaster funds, climate change, research, new technology, etc. [45].

In one study, researchers argued that grant funding allocation could be discussed regarding support for regional decision-makers and as a tool for national negotiation. Accordingly, three demand scenarios have been developed, showing how regional and nationally funded grants could be managed to reduce future gaps by comparing the results with current policies [46]. FinTech platform is one of these tools that can raise funds and donations, improve access to quality healthcare services, and help in financing by shifting the dynamics of payment plans and broadening financial access to healthcare services [47].

#### 2–2 Process

Cost information can be incorporated into a data-mining algorithm for each risk factor to estimate the budgets for providing health services for a specific target population [48]. Patient expenditure information and related analysis by AI can help pharmaceutical companies to optimize the medications manufacturing process and other industries for better inventory management. Apart from the healthcare domain, the proposed method could be used to predict weather and earthquakes and for several applications [49]. Prediction of the healthcare cost [50] and other significant variables in health financing, such as healthcare expenditure per capita (pcHCE) [51] or estimation of health expenditure [3] help the health system to have more knowledge-based policies. AI can facilitate the implementation of dynamic strategies for revenue maximization through demand learning, which might result in lower out-of-pocket and impoverishment costs. The proposed method balances the trade-off between exploitation (revenue maximization) and exploration (demand model estimation) [52]. Utilizing methods such as Multi-Criteria Decision Analysis (MCDA) could facilitate the development of a decision support system in healthcare, thereby contributing to the efficient, rational, and fair allocation of resources [53].

The potential use of machine-learning approaches in analyzing complex data can help informed decision-making, alleviate catastrophic health expenditures, and provide the needed budget [5]. Basic medical insurance does not protect households from catastrophic out-of-pocket (OOP) health expenditures. To reduce inequality, it would be beneficial to utilize big data tools and techniques to effectively screen poor households and strengthen the social and medical aid system for them [54–57].

#### 2–3 Context

AI can facilitate fraud detection in income tax data [58]. A recent ML analysis, the so-called K-Nearest Neighbour (KNN) model, can capture the self-predictive ability of the GDP and improve the performance of traditional time series analysis [59]. The AI-predicted GDP showed that factors obtained from real variables have much more impact than factors obtained from financial and price variables [60]. Related to the prediction of future econometric trends, one paper proposes an empirical mode decomposition (EMD) method designed to improve deep learning for understanding GDP trends and GDP data prediction research [61]. Another study examined the relationship between per capita GDP and total spending on health care. In particular, their analysis confirmed the growth theory through the indirect effects of healthcare expenditure on GDP [62].

Correlation between ideal retirement age and life expectancy [63], identifying the prevalence of pain and trends of pain associated with chronic diseases and personal out-of-pocket medical expenditures over time [64], and evaluating the association between low-value care with excess out-of-pocket cost are other AI-related analysis [65] that can be used by policymakers for advocacy to increase health revenues.

### 3- Pooling

#### 3 − 1 Context

In pooling, computational intelligence techniques are used to model the behavior of medical reviewers - professionals who assess whether medical requests should be allowed [66]. To make a more in-depth analysis of the relationship between public services and balanced economic development, one study constructs the measurement indicators of balanced economic development, public services, and local fiscal revenue and expenditure and empirically tests the endogenous relationship and influencing factors between them [67]. These findings can be used for fund pooling in territories and areas as local communities to improve access to healthcare for all. Additionally, another study has shown that economic growth positively enhances the quality and quantity of public service supply [67]. Further, costs related to increased cancer incidence and mortality rates worsen the loss ratio of cancer insurance products and create a financial crisis for insurers. Analysis of the same annual medical cost for the elderly compared to other ages can help risk pooling by preventive strategies [68].

#### 3 − 2 Content

AI can examine the choice environment of private online health insurance exchanges, which provide alternate venues for consumers to shop for various insurance plans. Theoretically, these estimates can help consumers identify the highest-value health plans [69]. OptiHealth is a recommender framework for selecting Pareto optimal health insurance plans. The recommender uses actuarial data to estimate the total annual cost for each plan and then recommends a small number of Pareto optimal plans [70]. Exploring the heterogeneity not just along socioeconomic variables but also according to some other characteristics of policy impacts can change the policy impact assessment from “whether the policy is effective” to “who the policy is effective for.” Therefore, the result has reference value for policymakers to formulate “optimal policy rules” and improve the qualification, re-design of the eligibility criteria, and subsidy standards of medical insurance [71, 72].

AI can also help the application of techniques for classifying the beneficiaries of an operator of health insurance, according to their financial sustainability, via their sociodemographic characteristics and healthcare cost history [66]. Identifying high-risk patients and the expense and indirect costs of implementing mitigation strategies helps better pool the health system. For example, financial toxicity mitigation tools resulted in financial navigators, charity aid, or insurance copay assistance programs. They guided shared decision-making concerning high-risk patient populations to align treatment decisions with their (spending) preferences and values [73].

Insurance companies need to convince their clients about the advantages they can receive, including direct monetary rewards through customized offers with lower premiums due to their healthy lifestyle choices; and/or indirect rewards such as coaching for well-being by using explainable AI techniques in the classifiers used by the risk assessment service. [25]. health programs could use AI to identify impactable high utilizers, possibly reduce inappropriate healthcare spending, understand better impactable healthcare conditions, and progress toward interventions to reduce inappropriate healthcare utilization [74]. AI methodologies can be applied in need-based and optimal insurance packages based on definite criteria. It will not only allow employers and insurance companies to design suitable insurance schemes for the provision of healthcare benefits but will also prevent financial losses in the long run [75]. Using clustering techniques in this field will also provide opportunities and solutions for decision-makers to monitor insurance coverage based on socioeconomic, geospatial, and demographic variables in general and health insurance in particular [76–78].

#### 3–3 Process

One study sought to develop and test a tool to accurately predict an individual’s risk of financial toxicity based on clinical, demographic, and patient-reported data before initiation of treatment, which resulted in a financial burden for the health system. Such analyses can help create a learning health system [73]. Related systems can provide risk assessment based on behavior for the health insurance sector [79]. To simplify the analysis of healthcare costs for the insurer and patient, the most important variables can be extracted to predict the healthcare insurance costs [80, 81]. For instance, chronic kidney disease (CKD) represents a heavy burden on the healthcare system because of the increasing number of patients, high risk of progression to end-stage renal disease, and poor prognosis of morbidity and mortality. One study aimed to forecast its prevalence through developing a machine-learning model that uses comorbidity and medication data. The model proposed in this study could be a valuable tool for policymakers in predicting the trends of diseases in the population, close monitoring of people at risk, early detection of diseases, better allocation of resources, and patient-centric management [82]. Finally, by AI applications, health insurance premiums can be predicted based on various parameters, such as age, gender, body mass index, number of children, smoking habits, and geolocation [83].

### 4- Strategic purchasing

#### 4 − 1 Context

The Internet of Everything (IoE) allows for the management and monitoring of numerous IoE nodes spread out across a smart city. This enables the support of various applications in different domains, including energy and resource management, intelligent transportation systems, and e-health [84]. New vectors can also be added to the health insurance packages to help the health and insurance sector construct mathematical risk equation models with parameters to map real-life risk conditions [85]. Resource allocation strategies can be developed for prioritizing limited healthcare capacity based on the computational characterization of spatiotemporal patterns of the disease transmission risks [86]. Developing a risk adjustment model of patient expenditures according to their vital signs, health status and lifestyle indicators, demographics, diagnoses, comorbidities, disease history, insurance, income level, and secondary diagnoses [87] have the potential to identify characteristic profiles of high utilizers of health care (85). This may help create clusters based on individuals’ demographic, economic, and health-related conditions and examine the clusters on future expenditures commonly used in healthcare utilization studies [88]. The design of injury prevention strategies [89] and allocation of resources such as GPs reflect the location-specific higher healthcare demand [90].

#### 4 − 2 Content

To improve resource management, cost containment, and responsibility in healthcare, it is crucial to establish information systems that can aid hospital managers in financial management, resource allocation, and activity planning [91–94]. The impacts of different operating strategies can be identified by building and validating computer simulation models, which leads to higher efficiency for the hospital’s resources without decreasing the quality of patient care [95].

Training the payment prediction model for individuals’ future payment behavior [96] or predicting coming days in the hospital using features extracted from customer demographics, past hospital admission, and hospital procedure claim data [97] can bring the benefits of providing references for medical management with specific diseases that could reduce the expense through effective control. This might reduce the cost of healthcare, which may decrease health insurance burdens in the future [98]. Data mining methods can be used to determine the key financial indicators in public hospitals, which might help improve their respective financial performances [99]. This decision support system also allows the government to predict preventive actions and resource allocation in the health sector [100].

Nationally representative physician data on their payment arrangements and practice characteristics can be used to create a parsimonious typology for incentive arrangements [101]. Supervised learning approaches, such as Random Forest, could substantially improve the prediction accuracy of counter-verification in PBF (performance-based financing) and thus increase the cost-effectiveness of verification [102]. The utilization of big data can enhance the accuracy and promptness of information in infrastructural healthcare Project Finance (PF). The collaboration between public and private partners, facilitated by networking big data and interoperable databases, can increase the creation of value, leading to better value for money and lower risk. Big data can also simplify supply chain processes, widen economic opportunities, and facilitate sustainable planning for intelligent healthcare investments [103]. AI can facilitate cost-benefit analysis, which will contribute to the efficiency of human resources strategies to demonstrate the return on investment [104].

The results of AI analysis can offer valuable guidance for policymakers to address the uneven distribution of medical resources, enhance regional public health systems, and facilitate government coordination in allocating medical resources at various levels. This can ultimately improve the overall performance of the medical and health service system and promote its balanced and coordinated development [105]. However, these findings must be refined and customized to suit specific diseases. One study introduced a tool to estimate the disease burden for the population and health system. These tools can also create regional snapshots focusing on particular populations through national and regional public health policies [106]. Finally, AI can help the primary healthcare network (PHCN) to develop monitoring systems and resource allocation monitoring [107].

#### 4 − 3 Process

Insights for insurance industries or individuals to understand the magnitude of healthcare fraud within different healthcare systems help design better strategies to combat fraud [108]. AI also helps to model the detection of abusive utilization patterns in various payment arrangements [109].

The length of hospital stay or the magnitude of medical expenses can also be used for cost-effective analysis [110]. The frequency and patterns of prescriptions used for treatment [111], safety rate of treatment interventions regardless of age [112], and risk asses of return visits [113] guide to reevaluating and providing effective interventions.

Morbidity Related Groups (MRG) is a new system, adapted from the Diagnosis-Related-Group-System (DRG), which has been developed to control expenditure through the classification and compensation of hospital cases [114]. One study aimed to identify super-utilizers and low users; an established technique was used to categorize patients into four expenditure groups [115]. In another study, researchers sought to anticipate variations in patients’ medical expenses for the following year and pinpoint significant factors contributing to this forecast. They focused on the impact of pharmacotherapy and other medical aspects, such as hospitalizations and visits to outpatient physicians [2]. Additionally, AI has the potential to enhance resource allocation decisions by providing a more efficient alternative to a hospital’s current approach that employs a DRG grouper [116].

AI capabilities for e-Health and digital health provide a powerful forum for debate on cost-effective resource allocation [117]. AI can transform the existing healthcare delivery models by inducing a redesign of public accountability systems and the traditional relationships between professionals and patients. The outcome will be a transformative change integrating digital technologies into public sector modernization. AI has a great potential to empower patients while improving their health, personalized experiences, and individual well-being [118]. The “smart” financial service model in the era of AI has also played a profound and revolutionary role in the reform of financial operation mechanisms [119]. In one study, researchers developed two financial road maps for the low performer hospitals to improve their financial performance [120].

The architecture of a system developed for the Polish National Health Fund (NHF) branch has been introduced in one study. The experience with controlling system modeling can be used to extend the developed model by new data structures, relations, inference rules, tools for result data visualization, and implementation of solutions to the contextual data analysis [121]. Another study investigated the accuracy of laboratory tests, improved patients’ safety, and reduced unnecessary costs associated with wrong orders [122]. One study associated the link between the rate of oral antibiotic prescriptions and the diagnosis of infectious diseases [123]. Predicting successful visit payments, helping to identify patients in need of financial support, allowing for the development of customized and patient-oriented payment plans, assisting the patients in benefiting from state-funded programs and navigating through the network restrictions, out-of-pocket costs, and confusion surrounding healthcare costs and billing systems [13] are among other applications of AI to improve patients’ satisfaction. By providing a clear picture of the insurance options, patients can apply for affordable health insurance or state-funded programs and overcome obstacles to enrollment [13].

## Conclusions

This paper discussed a number of AI applications in health financing, including governance, revenue raising, pooling, and strategic purchasing of health financing. To address health problems related to financing, AI applications should be established and improved. In addition to providing insights into complex data sets that would be difficult or impossible for humans to analyze, artificial intelligence can transform health system financing, processes, and policy-making. Despite the challenges associated with implementing AI in healthcare systems, such as maintaining patient privacy and ethical standards, its benefits are evident. To improve outcomes in health financing, we encourage policymakers to continue exploring ways to incorporate artificial intelligence into their decision-making processes.

The potential of AI is enormous, and methods should be developed outside of the finance industry. Future research can concentrate on examining actual health finance data, including the inclusion of beneficiaries and looking into the effects of weighting various variables. It would be beneficial to investigate AI applications on other health building blocks. In order to confirm the efficacy of the various strategies proposed in this study, real-world data or simulated data can be used for testing. The impact of AI technology on financial issues, management and strategic aspects of it, and how it might be implemented in other health building blocks all require cross-disciplinary studies.

The structure for initiatives to encourage sustainable financing can be provided by AI. Providing health services and initiatives might be given priority by politicians to ensure financial sustainability while simultaneously achieving health goals. The results show that AI applications can be considered as a new paradigm in health financing, which can be added to the development literature.

The present study has several strengths that contribute to the field of AI applications. First of all, the findings of this future study will shed light on how AI might be used to improve health financing. Our research discussed a sizable number of AI applications, offering insightful information for future capacity development and sustainable policy creation. Our study’s findings can be used as a foundation for using sophisticated models, which is a novel addition to the body of literature. We are hopeful that our work will help scientists choose and use these models and tools efficiently. In particular, our study can offer a starting point for future research on AI techniques to investigate complicated relationships between numerous components or design a platform that can be used by health systems to manage health financing.

The absence of negative impacts and ethical issues pertaining to technological breakthroughs, which would have offered useful insights, is one of the study’s limitations. It would have been beneficial to investigate AI applications in every aspect of health financing from different perspectives. Finally, our study did not examine the potential of AI in other health building blocks and solely concentrated on the finance area.

## Data Availability

Not applicable.

## Abbreviations

AI: Artificial Intelligence
GDP: Gross domestic product
ML: Machine learning
GFATM: Global Fund to Fight AIDS, TB, and Malaria
